# Analyzing the effects of a chemical peel on post‐inflammatory hyperpigmentation using line‐field confocal optical coherence tomography

**DOI:** 10.1111/srt.13496

**Published:** 2023-10-05

**Authors:** Shazli Razi, Tricia Mae Raquepo, Thu Minh Truong, Babar Rao

**Affiliations:** ^1^ Rao Dermatology Atlantic Highlands New Jersey USA; ^2^ Department of Internal Medicine Jersey Shore University Medical Center Neptune New Jersey USA; ^3^ Center for Dermatology Rutgers Robert Wood Johnson Medical School Somerset New Jersey USA; ^4^ Department of Dermatology Weill Cornell Medicine New York New York USA

Dear Editor,

Line‐field confocal optical coherence tomography (LC‐OCT) is a burgeoning imaging device that allows for real‐time, high‐resolution, in vivo assessment of skin.^1^, ^2^ LC‐OCT utilizes a laser to measure the backscattering of light from cellular structures.^3^ LC‐OCT can visualize up to 500 μm at quasi‐histological vertical (optical coherence tomography) and horizontal (reflectance confocal microscopy) views of the skin to produce a three‐dimensional image. In comparison to traditional confocal microscopy, LC‐OCT allows greater depth of visualization, and faster image acquisition.^1^, ^2^

According to the 2020–2021 Aesthetic Plastic Surgery National Databank Statistics, chemical peels are one of the most extensively performed non‐invasive cosmetic procedures in the United States.^4^ Chemical peels can be used to treat photoaging, lentigines, melasma, and post‐inflammatory hyperpigmentation.^5^ Chemical peels cause controlled chemical injury leading to remodeling, regeneration, and wound healing.^6^ We present a case study in which the patient presented to the clinic with complaints of hyperpigmented spots on his limb. To treat post‐inflammatory hyperpigmentation, the patient underwent two treatment sessions of targeted spot treatment using a commercially blended chemical peel containing trichloroacetic acid and lactic acid, with a 1‐month interval between sessions. LC‐OCT was utilized for imaging before and after completion of two treatment sessions. Images were acquired in a protocol to obtain vertical, horizontal, and 3D images. During the analysis of the images in vertical and horizontal views, the “deepmelanin” algorithm was used as it is the recommended algorithm for pigmented lesions. The algorithm was utilized to brighten melanin/pigmentation.

The analysis of pretreatment vertical images depicts hyperreflective pigmentation scattered through the epidermis. Post‐treatment vertical images show that pigmentation has shifted upwards in the epidermis. As a result, the upper layer of the epidermis appears brighter, and the lower epidermis displays reduced reflectivity (Figure 1). Furthermore, pre and post‐treatment horizontal (confocal) images of the epidermis were analyzed at the same depth. Pretreatment horizontal images demonstrate the presence of extensive areas of brightness indicating abnormal pigmentation. Conversely, post‐treatment horizontal images display a notable decrease in hyperreflective area, indicating a reduction in pigmentation at that specific depth (Figure 1). This reduction suggests that melanin has migrated upwards. This finding aligns with the results of our previous study that examined the effects of chemical peel on melanin using reflectance confocal microscopy.^8^ In the current study, a 3‐dimensional (3D) projection mode was used in max reflective setting, to compare pre and post‐treatment. Hyper‐reflective parts of the image may represent a combination of pigmentation (melanin), pigmented cells (keratinocytes, melanocytes), and inflammatory cells. The pretreatment images show presence of hyperreflective pigmentation dispersed through epidermis, corresponding to greater melanin concentration. However, post‐treatment 3D projection images demonstrate an upward shift of pigmentation resulting in hyperreflective stratum corneum on the surface but a markedly reduced hyper reflectivity as we analyze deeper layers (Figure 2). On clinical examination, the hyperpigmented spots appeared lighter, fragmented, and less prominent after treatment sessions. The clinical findings align with the LC‐OCT findings in vertical, horizontal, and 3D modes.

**FIGURE 1:**
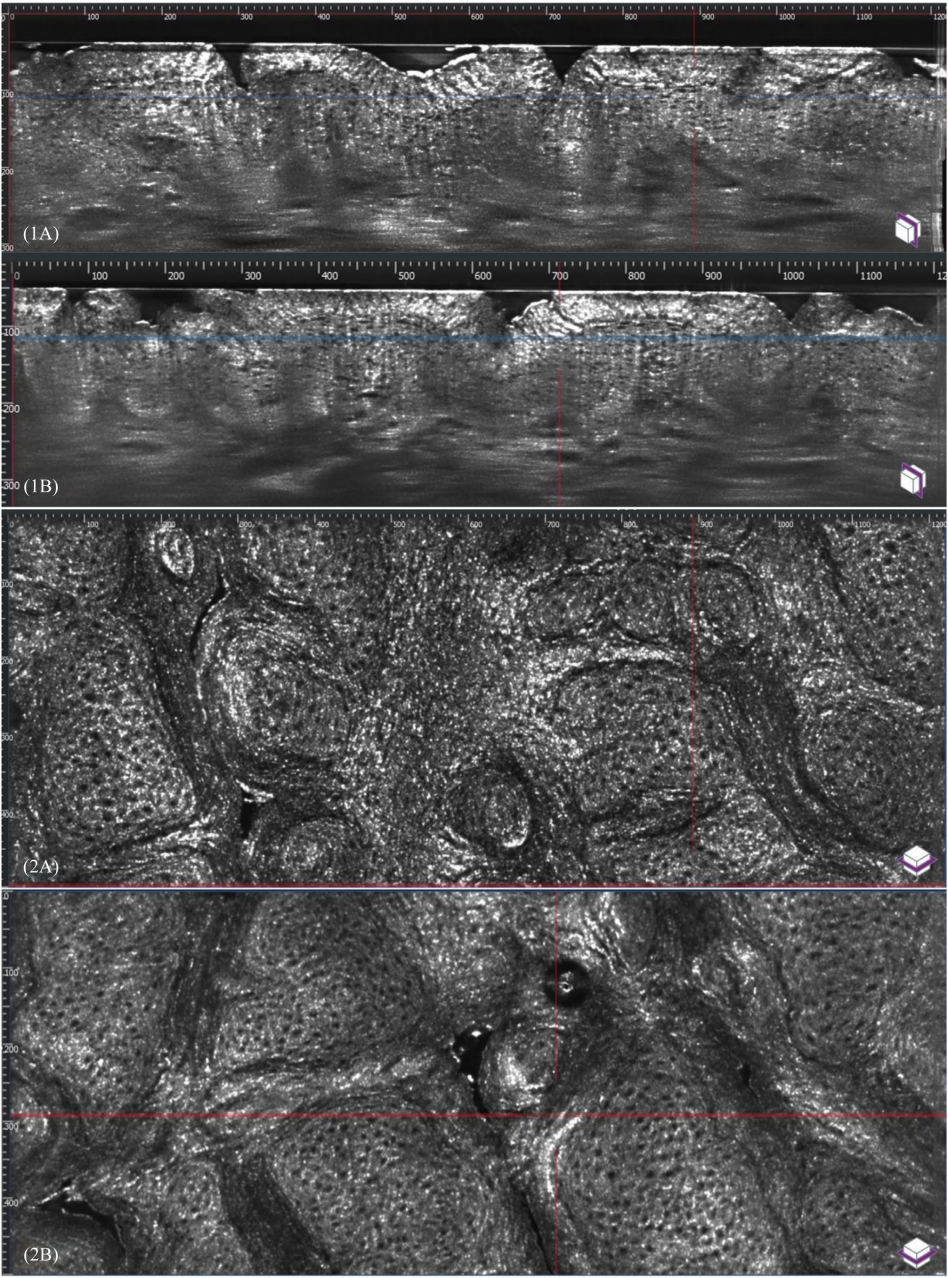
Vertical or optical coherence tomography view of LC‐OCT (1A) Pre‐treatment. (1B) One month after second treatment session. Horizontal or Confocal microscopy view of LC‐OCT. (2A) Pre‐treatment. (2B) One month after second treatment session.

**FIGURE 2:**
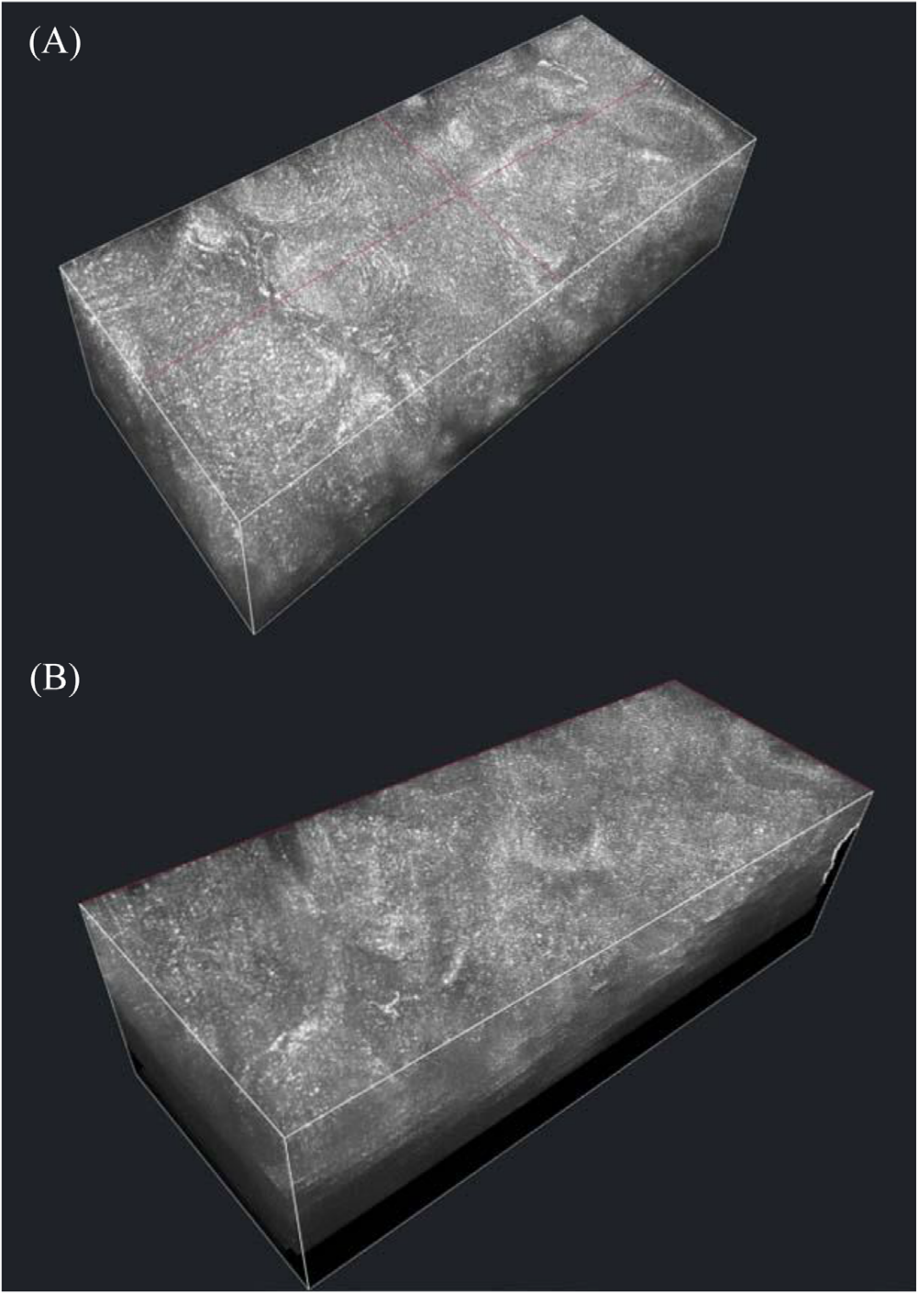
3‐Dimensional projection view (max reflectivity) of LC‐OCT. (A) Pre‐treatment. (B) 1 month after second treatment session.

Optical imaging devices, such as LC‐OCT, that allow in‐vivo subsurface evaluation of the skin have the potential to positively impact research in dermatology. These devices will enable clinicians to evaluate the effectiveness of cosmetic procedures and products with enhanced precision.

## Data Availability

The data that support the findings of this study are available on request from the corresponding author. The data are not publicly available due to privacy or ethical restrictions

## References

[1] Ruini C , Schuh S , Sattler E , Welzel J . Line‐field confocal optical coherence tomography‐Practical applications in dermatology and comparison with established imaging methods. Skin Res Technol. 2021;27(3):340‐352. doi:10.1111/srt.12949 33085784

[2] Dubois A , Levecq O , Azimani H , et al. Line‐field confocal optical coherence tomography for high‐resolution noninvasive imaging of skin tumors. J Biomed Opt. 2018;23(10):1–9. 10.1117/1.JBO.23.10.106007 30353716

[3] Ogien J , Levecq O , Azimani H , Dubois A . Dual‐mode line‐field confocal optical coherence tomography for ultrahigh‐resolution vertical and horizontal section imaging of human skin in vivo. Biomed Opt Express. 2020;11(3):1327–1335. 10.1364/BOE.385303 PMC7075601 32206413

[4] Procedural Statistics [Internet] . The Aesthetic Society. [cited 2023 May 30]. Available from: https://www.theaestheticsociety.org/media/procedural‐statistics

[5] Rendon MI , Berson DS , Cohen JL , Roberts WE , Starker I , Wang B . Evidence and considerations in the application of chemical peels in skin disorders and aesthetic resurfacing. J Clin Aesthet Dermatol. 2010;3(7):32–43. PMC2921757 20725555

[6] O'Connor AA , Lowe PM , Shumack S , Lim AC . Chemical peels: a review of current practice. Aust J Dermatol. 2018;59(3):171‐181. 10.1111/ajd.12715 29064096

[7] Breugnot J , Rouaud‐Tinguely P , Gilardeau S , et al. Utilizing deep learning for dermal matrix quality assessment on in vivo line‐field confocal optical coherence tomography images. Skin Res Technol. 2023; 29: e13221. 20221110. doi:10.1111/srt.13221 36366860 PMC9838780

[8] Razi S , Bhardwaj V , Ouellette S , et al. Demystifying the mechanism of action of professional facial peeling: In‐vivo visualization and quantification of changes in inflammation, melanin and collagen using Vivascope® and ConfoScan®. Dermatologic Therapy. 2022;35(11):e15846. 36129212 10.1111/dth.15846 PMC9787425

